# *‘You’re out on a limb*, *on your own’*: Social care personal assistants’ (PAs’) reflections on working in the Covid-19 pandemic ‐ implications for wider health and care services

**DOI:** 10.1371/journal.pone.0295385

**Published:** 2023-12-27

**Authors:** Caroline Norrie, Olivia Luijnenburg, Jo Moriarty, Kritika Samsi, Jill Manthorpe

**Affiliations:** NIHR Policy Research Unit in Health and Social Care Workforce (HSCWRU), The Policy Institute, King’s College London (KCL), London, United Kingdom; Yeungnam University College of Pharmacy, REPUBLIC OF KOREA

## Abstract

**Context:**

In England, Personal Assistants (PAs) are part of an international trend towards state funded but client-hired or directly employed care workers. The Covid-19 pandemic highlighted and exacerbated pre-existing risks and advantages of this arrangement for both PAs and people with care and support needs.

**Objectives:**

We aim to report PAs’ reflections on their experiences of working since the pandemic started in 2020 and highlight the longer-term implications for health and care services.

**Methods:**

We undertook a large-scale, qualitative study in 2016–17 involving interviews with 104 PAs about their working lives. We re-interviewed PAs from this group twice to ask how the pandemic had affected them, once at the start of the pandemic in Spring 2020 and again in December 2021 –April 2022. This article reports findings from the last set of interviews undertaken with 38 PAs. Thematic analysis was conducted of interviews in which PAs discussed changes in tasks and responsibilities, pay and conditions, training, relationships and plans.

**Findings:**

This article focuses on the following themes: PAs’ perceptions of their outsider status; support and training needs; job security; and whether PAs have an appetite for regulation to provide greater professional standing and connections.

**Limitations:**

Interviews in this study were carried out during the Covid-19 pandemic over the telephone or virtually rather than in person so may have missed certain body language or informal relationship building. The sample may be under-representative of non-British PAs. We were unable to triangulate participants’ accounts with others’.

**Implications:**

This study highlights the importance of national and local government including the PA workforce in planning for national emergencies. Consideration should be given by policy makers and local health and care systems to how PAs can be better supported than currently.

## Background

‘Consumer-directed care’, ‘cash for care’, ‘self-directed care’, or personal/direct payments are terms used internationally to refer to state funded transfers of money to individuals in need of care (or their family) so they can organise their own support. These self-directed care schemes have been a growing part of social care provision internationally since the 1990s [1–4]. Consumer-directed care can be viewed as part of ‘care in the community’ or ‘care at home’ policy movements [5]. It is potentially becoming more significant due to the Covid-19 pandemic reducing the willingness of people to move into care homes [6].

Local authority (LA) Direct Payments (DPs) and later Personal Budgets (PBs) were introduced over 25 years ago (Department of Health (DH) 1996) in England; they enable eligible individuals with care and support needs (or their family) to employ a PA, rather than the LA providing services through a care agency. These approaches are celebrated for the greater choice and control they offer to individuals, but, at the same time, care workers employed under this model have found themselves stranded on the fringes of the social care workforce during the pandemic [7]. PAs are very often unknown to an LA, have no managerial oversight, and can be viewed as isolated practitioners [8].

PAs as a group in England are unregulated and also unrepresented by a professional association. They are not required to undergo criminal record checks via the Disclosure and Barring system (although the LA may pay for this if requested) despite working with adults who may be vulnerable and being in receipt of public funds [9]. Some PAs are in contact with a brokerage agency which may assist some potential PAs and employers in contacting each other and organising administrative processes such as insurance cover and payroll [10].

Skills for Care [11] estimates that of the approximate 220,000 people (including family carers) receiving direct payments in 2021/22, an estimated 65,000 (29%) of these recipients were directly employing their own staff. It should be noted some people also employ PAs via other funding streams or by using their own funds. People with care and support needs are referred to as *employers* in this article although many PAs are self-employed.

The Covid-19 pandemic highlighted existing tensions around the regulation and monitoring of PAs, for example, risks of harm, exploitation or abuse of both PAs and employers [12–14]. Furthermore, it exposed the importance of PAs as a group who need to be included in infection control and disaster planning. In England, risks to social care were realised late, with the National Health Service (NHS) being prioritised over community settings [14–16]. Against this background, PAs were not generally mentioned in national policies. Guidance for PAs and their employers confirming their eligibility for Personal Protective Equipment (PPE) and free flu vaccines was not issued until November 2020 [17], several months into the pandemic.

Finally in England, the cost-of-living crisis and labour shortages (created in part by Brexit–the withdrawal of the UK from the European Union), can be viewed as creating another level of risk for health and care systems [18]. Skills for Care [10] reported that the number of vacant posts in adult social care had increased by 52% in one year standing at the highest rate since records began in 2012/13. The average vacancy rate of PAs was 13.1% which was higher than the vacancy rate amongst care workers (9.8%) as at February 2022. An online survey of 983 people employing PAs found 77% of people needing to recruit and retain a PA were finding this hard to do and this had worsened during the pandemic, with low pay, poor terms and conditions, and insufficient hours identified as contributory factors [7].

We undertook a large-scale, qualitative study about the working conditions of 105 PAs in 2016–17 to gain their perspectives. Participants had been initially recruited predominantly through disability organisations, and had to be currently working as a PA. The first study focussed on PAs’ work, pay and conditions, training, and multi-agency working [19–21]. We re-interviewed 41 of this group for a second time between April and May 2020, at the start of the Covid-19 pandemic about how their working lives had been impacted [22, 23]. We returned to this cohort for a third time from December 2021-April 2022 to explore the longer-term implications of the pandemic and how they were affecting PAs’ working lives. This article reports findings from this third set of interviews.

## Methods

### Objectives

The aim of this study was to find out what enduring impacts the Covid-19 pandemic has had for PAs and the nature of their working relationships with their employer, their employer’s family (where relevant) and other community-based health and care professionals.

Secondary objectives were to explore:

1. What has happened in PAs’ working lives since the height of the pandemic?
2. What has changed, if anything, in PAs’ working relationship with their employer(s), their employer’s family (where relevant) and community-based professionals, and in terms of their employment conditions?
3. How do PAs envisage their future working lives in a ‘post-pandemic’ world–and whether / how the pandemic has shaped working plans?

All 105 PA participants from the original study had given permission to be recontacted and were invited by email or letter to take part in this present study. We sought to establish whether individuals were still working as PAs or were now in different employment and aimed to interview any PAs who were no longer working in the role to ask why.

PAs who expressed interest in participating in the study were emailed a participation information sheet (PIS) and consent form. Interviews were arranged at a mutually convenient time, were conducted by telephone or a video-conferencing platform and lasted between 30–60 minutes, where possible with a researcher whom they had spoken to previously. Participants were interviewed in their home setting and apparently on their own. Each participant was offered a £20 gift voucher in appreciation of their time. Interviews were recorded, with permission and transcribed by a private company with whom the researchers have a confidentiality agreement.

Interviews were conducted by three experienced researchers (OL, CN (females) MS (male)) whose backgrounds included LA research, social care, gerontology and anthropology. Other than OL all the research team had undertaken research on this subject previously and are experienced in care workforce research. Researchers asked questions on a range of topics following an interview schedule which covered experiences of working, including tasks and responsibilities, during the pandemic, relationships with employers/families, pay and conditions, training and plans.

Framework analysis [24] was used to thematically analyse interviews and to focus on phenomena of interest taking a phenomenological approach. Analysis was undertaken of the transcribed interviews using NVivo (version 1.6.1) to manage the data [25] with data being coded (by MS, OL and CN) following a framework based on the interview schedule combined with emerging free codes. Initial findings from the interviews were presented to the HSCWRU, King’s College London Patient and Public Involvement and Engagement (PPIE) group (members consist of service users, carers, and practitioners including an LA professional with experience of working with PA brokers), who shared reflections on personal experiences in relation to our emerging themes and potential areas for analysis. Emerging findings were not shared with the participants but all those who wished for a copy of the findings were sent one. The research team met online regularly to discuss study progress, emerging findings and ensure consistency of coding. Data were interrogated further using key word automated text searches within NVivo as part of the coding process.

Ethical approval was granted by King’s College London [Ref: MRA-20/21-24821]. Participants were informed in the participant information sheet and verbally that data would be treated as confidential and that findings would be reported anonymously. Written or oral consent was obtained from all participants. The research team prepared for the possibility of the interview causing distress to PAs or of safeguarding concerns being reported and had protocols in place for such eventualities.

This article focuses on the longer-term implications for health and care systems of PAs’ reflections on their work during the pandemic and plans.

### Findings

Thirty-eight participants were recruited from across England (see Table 1), the majority being White, British, middle-aged women. Pay per hour ranged from £9–20 (mean £12.69) with seven of the 38 participants receiving £16 per hour or over. Eight participants had children living with them. Participants worked a range of hours per week, ranging from three to 40. Many participants had other jobs alongside their PA work. The description of this workforce chimes with other studies; although a Skills for Care (2022a) survey of 2,228 PAs found a much higher number of PAs who were employed by a family member (55%) than in our sample, where only one PA was in this situation.

**Table 1:** The sample.

Gender	32 females, 6 males
Age	Range from 26 to 75;​ most in 50s and 60s
Ethnicity	35 White, 1 Mixed, 2 Asian
Nationality	35 British, 1 Romanian, 1 French, 1 Polish
Hours worked per week	3–40 hrs
Pay	£9 to £20 per hour; mean £12.60 per hour
Other jobs alongside	Care home worker, therapist, examination invigilator, shop worker, prison worker, counsellor, charity administrator, retired health/social care professionals
Post-Covid-19 working situation	28/38 still working as a PA with no imminent plans to retire (including 2 working very reduced hours and now predominantly working in more secure employment/less Covid-risky roles)
	5 Retiring (generally only working with one employer now) 5 No longer working as a PA (3 due to own or a family member’s vulnerability)

The themes that emerged relating to longer term impacts of Covid-19 on the PA workforce and implications for health and care systems were: PAs’ perceived outsider status; PA support and training needs; PA job insecurity; and whether PAs have an appetite for greater regulation to provide greater professional standing and connections. It should be noted, these aspects of the PA role are not new themselves but our focus on the effect of the pandemic on them, and implications for future planning have been less addressed.

### PA outsider status ‐ ‘we need to be more recognised’

PAs reported feeling unknown as a workforce, at a national and local level, despite being funded by public agencies. As the following PA described, there is a lack of understanding of the role itself:

*It’s almost like they weren’t aware that people were even doing this job*. *It was maddening*. *The amount of people we spoke to was*, *‘Well*, *if you’re a carer you work in a care home’*. *‘No I don’t’*. *‘Well*, *you go to people so you’re employed by a company*.*’ ‘But I’m not employed by a company’*. *I was having to explain my job to them before they could even explain how or why I was affected or not*. *You know*, *it’s just—it sometimes makes you feel like you’re out on a limb on your own*. *And you’re doing everything with no thanks*, *you know*? *It’s not nice*. *Not nice*. (PA 107)

PAs reflected on feeling overlooked and undervalued despite the importance of their work during the pandemic:

*I think just remember that we’re there*. *You know*, *it was all the focus was on*, *was on care homes*, *you know*. *If I didn’t take the initiative*, *God knows when I would have been vaccinated*. *Again with the PPE (Personal Protective Equipment)*, *it was all what was left was sent to care homes*, *and even that was minimal*. *You know*, *we were left to fend for ourselves*. *But at the end of the day*, *the client couldn’t*, *for a lot of people especially those that lived on their own*, *if they didn’t have the PA come in they wouldn’t have been able to [pause]…*. *to live*. *[] But for the government we were*, *we could have just been filed away as non-essential*. (PA 119)

PAs indicated they wished to be known to their LA, communication established or improved, and called for a point of contact for them in health or care agencies such as the LA. It was no longer sufficient for their employer to have these connections as they might not be in a position to contact their funding source and in situations such as a pandemic the pattern of reviews by the LA of direct payment recipients and other service users had generally been suspended. Some noted that while freedom from any management or external control had previously seemed a liberating aspect of the job, the pandemic exposed this as potentially problematic:

*I think there should be a*, *they should have somebody*, *a point of contact supporting people saying*, *‘Do you know what*? *You’re doing a great job*.*’ (…) but you don’t get anything like that*. (PA 107)

There were exceptions to this situation however and the following quote outlines the support one PA received through a brokerage agency, a voluntary sector group offering a service to PAs and disabled people that was funded by their LA:

*PA11*: *I’ve got a letter*. *I’ve never had to use it*. *But I’ve got a letter that I could go and get the PPE myself if I want*.

*[] I* *: Do you know who sent you that?*

*PA11*: *x Council*. *It says that I can access masks*, *gloves*, *aprons*, *hand gel as well*.

*I* *: Because you were PA?*

*PA11*: *Because I was considered a carer*, *yeah*.*[] I’ve lost the letter now*, *but I think it’s to do with direct payments*. *It goes through the council to some extent*, *but it also goes to a voluntary agency that does the payroll*. *So they updated the council with the list of personal assistants or whatever and I think that’s how I got my letter*.

Those PAs who were in contact with brokerage agencies or reported direct links with LAs generally reported feeling much more supported as the following PA illustrated:

*Myself and my clients appreciate the fact that we*, *because of our membership*, *[brokerage agency] had to meet certain standards*. *And we have a lot more training than people from different agencies*. *We’re a lot more knowledgeable*. *I think it’s very beneficial*. *And I would encourage anybody to go and join a scheme like it*. *(PA104)*

A minority of PAs expressed the opinion that the pandemic had meant health and care systems were more aware of PAs, for example one thought this was evident in their newly recognised entitlements to free flu vaccines from the NHS:

*And for all the years working for my employer*, *we did get the flu vaccinations every year*, *and we’ve always paid for the flu jabs*. *last year when I wanted to get my flu jab it said on a list*, *Personal Assistant which I was surprised*, *actually*, *that we finally kind of were acknowledged [laughing]*. (PA 23)

### PA support and training needs ‐ ‘*PAs just get thrown in and told to just work it out’*

PAs discussed how their work changed during the pandemic and whether they felt supported. There was great variety of experiences across the sample. Most PAs at the beginning of the pandemic had been asked by their employers not to come to their homes during lockdowns, or to reduce their hours to protect employers; other PAs stated their work routines had stayed the same and for some, work requests increased as families could no longer visit employers and so PAs took on additional responsibilities. One PA reported that their employer’s family member had newly been permitted to act as their relative’s PA by the LA to provide income and continuity of care.

PAs reported needing assistance with payment arrangements during the pandemic. A range of experiences was recounted ranging from whether PAs received pay if they were unable to work in lockdowns or if self-isolating. Some PAs received furlough payments (government payments if unable to work) if they were self-employed, other continued to receive pay via LA funded brokerage agencies. Some PAs who received pay directly from their employer or family reported that payments immediately stopped at the start of the pandemic. In other cases, families reduced PA hours, but made up the difference in their pay. One PA reported that their pay stopped overnight having worked for a family for ten years. The following quote illustrated the difficult financial situation faced by many of the PAs:

*There was*, *right at the very*, *very beginning*, *so when we first went into lockdown*, *[*..*] there was kind of like a month and a half where I didn’t get paid*, *except for the hours I’d worked*. *But then I was back paid […] the brokerage company was allowed to back pay those hours*. *But obviously*, *you know*, *if I had lived on my own*, *[] with my mortgage […] I would have been well and truly screwed*. (PA 119)

PAs also reported a lack of knowledge and support about access to state sickness pay if they needed to self-isolate themselves. PA 52, for example, discussed lack of support from the brokerage agency (whom she referred to as a company) used by her employer when she inadvertently apparently transmitted Covid-19 to her employer and went on sick leave:

*But the manager at work*, *she was a new manager and she was absolutely awful*. *She was really—I just can’t bear to talk to her now because she just said*, *‘that it was my fault that I took it in the house’*. *I thought there was no support*. *There was nothing at all for me*. *And I was off work*. *And then the girl I look after*, *the company sent her like a get-well package*. *I didn’t even get a phone [call]*. *I didn’t get nothing at all*. *It was just like*, *I felt like a leper*. (PA 52)

A major narrative of the interviews was the stress of the increased risk and accompanying responsibilities of keeping employers safe during different phases of the pandemic. Some PAs reflected on the lack of access to wellbeing support to meet their mental health needs during this time, with several suggesting the need for a group or peer mentoring scheme:

*It’s because the work is really responsible*, *even more when you’re working with extremely vulnerable person*. *I think PAs have to start looking into [] taking care of their own mental health as well*. *Especially that in many places where PAs work*, *they work one to one*. *They don’t have other PAs to back you up*, *to have your back*, *to talk to*, *because then no one else know your work environment and knows your situation*. *[] Definitely the Covid situation*, *it really put a lot of stress on us all*. *Yes*, *so I think looking after yourself as a PA*. *Yes*. (PA 23)

One severely affected PA who had been hospitalised with Covid-19 explained how this impacted on her work plans:

*I’m alright but my anxiety is bad*. *I don’t like going crowded places*. *I do struggle*. *I was really*, *really poorly*. *I was very lucky [hospitalised]*. *[] It affects*, *obviously*, *my job because I hear the word Covid and I panic*. *I start panicking again*. *And it’s a serious situation []*. *Well I can do it*, *but I really struggle with it*. *Like if you want to go to the community and watch a show with your clients and that*, *I really struggle with that kind of environment*. *I’ve had a massive trauma*. (PA 107)

In contrast to our previous studies, PAs recounted a range of training opportunities now available to them; many discussed annual refresher courses in manual handing, first aid and infection control training. New to this study was mention of the range of online training opportunities that were now available to PAs via their LAs, broker agencies, or public webinars.

This PA (PA 11) was enthusiastic about her experiences of support through broker companies and was relishing new online training opportunities:

*[Named council]*, *offer everything*. *The amount of things that you can pick up and do*. *I’ve had three or four courses that have come through today if I want to do them*, *infection control and whatever*. *There’s specific Covid courses*, *we’ve done them at work*. *Ours are online*. *I like face to face because of course you can’t do face to face*. *But there’s plenty of Zoom or Teams things that you can do if you know what you’re doing []*. *[] [Council x] No*, *I just think they’ve been absolutely amazing*. *If there’s anything that I needed*, *they would help me find it*, *I’m sure*. (PA 11)

Also new were mentions of the deteriorating mental health of some employers which they attributed to the pandemic generally, although others discussed other difficulties; dealing with abusive behaviours was cited as a training need by several. A couple of PAs, for example in the quote below, mentioned enjoying undertaking more nursing tasks due to Covid-19 which reflects our previous study which found PAs potentially willing to take on more nursing tasks (20).

*I’ve had a few 111 (following advice from phoning a NHS helpline) cases where I’ve had to take clients in [to hospital from at home] with Covid*. *Like one of the three [employers] that got it*, *I wasn’t happy with his breathing and his oxygen*, *so I learned*, *I was taking oxygen levels every day*, *I became like a mini nurse*, *and I really liked that role of that care because it was serious care [] One of them [employers] I wasn’t happy with and he ended up in hospital*. *I went into hospital with him because you can’t leave—that was the only thing*, *when I went to hospital*, *there was nobody there from learning disabilities to take over from me*, *and you can’t leave them on their own*. (PA 12)

But instances of PAs being isolated and lacking training opportunities about COVID-19 were frequently recounted, for example, in the comparison below about greater professional development being possible in care homes:

*[Care home workers] they got a lot more hands-on experience and training because it was simple stuff like when you’re washing somebody*, *stand to the side of them as opposed to face on [to avoid spread of Covid] […] I wouldn’t have learnt that if I hadn’t talked to my friends in the care homes*. *[…] PAs just get thrown in and told to just work it out*. *[] If somebody came to me and they were new to caring*, *I’d just tell them to leave*. *Go find a nice little job somewhere else*. *[] I just wouldn’t recommend the profession because it’s not enough for me* … *there’s not enough training*. (PA 11)

### PA job security–‘there’s not enough of us’

Within our sample, 28 out of 38 participants were continuing to work as PAs with no imminent plans to retire or leave the work (see Table 1). Of the original sample (104 PAs) only 38 individuals replied to the invitation to participate in this study; it is unknown if those who did not respond were no longer working as PAs, and it is possible we interviewed particularly committed PAs.

Nonetheless we interviewed five PAs who were no longer working in the role. Three of them felt they could not continue in the role because of increased risks of Covid-19 on their own or a family member’s pre-existing health conditions. One PA had moved abroad, and another had moved into a professional role. Another PA had moved to work in a care home to ensure greater job stability, while the PA quoted below had found new employment in another sector and reduced her three PA employers to one, due to fears around contracting and passing on Covid-19 and to improve her job stability:

*I made the choice*, *obviously*, *to change into something [new job] just a little bit more stable and it’s not much safer but [] it was getting a bit too much*, *because I had three women that I used to visit and be a PA for*. *Well*, *it just a got little bit ‐ obviously*, *it got quite scary at some points with Covid*. *And I just thought*, *going from one person’s house*, *who has maybe like*, *two or three other carers next to me*, *and then go into the second person’s house who’s got the same situation*, *and then I go to the third one*. *I didn’t feel safe*. *[] It’s not the sort of job that you can social distance*. *You can’t help them get washed from the other side of the room*. *So it was more me*. *I decided that I wanted something to give me a full wage*, *but obviously wanted to keep the one lady because I’m quite fond of her*. (PA 11)

Other PAs mentioned poor pay, especially, given the cost-of-living crisis, as a reason for leaving the role:

*People are going to reconsider whether they can continue to work in this area*, *and again*, *it’s not because they’re not committed*. *It’s not because they don’t put their heart and soul into their work*. *But it’s not an affordable—it’s not what people do for*, *you know*, *you hear NHS nurses saying they don’t*, *you know*, *they don’t work in the NHS to get rich*, *or they don’t do nursing as a profession to get rich*. *But it’s even worse for care workers*, *and in lots of ways there’s*, *you know*, *there’s more isolation*. *There’s more*, *kind of complexity being out in the community*. *So*, *yeah*. *And I think there needs to be a huge rethink in terms of how things are structured in the care sector*. (PA 31)

The quote below highlights one participant’s assessment that PAs are leaving the role due to stress:

*It’s just all been a bit of a mess*, *hasn’t it*? *Not knowing what to do and when*. *I think really the scheme that we’re accredited to*, *I think they need to take into account that PAs over this time have really kept the care system running—[] And I think they need to look at how we can be supported and if there’s any problems that people have*, *they want to give up the job because they can’t cope with it anymore*, *and*, *you know*, *I think that needs to be acknowledged*. (PA 01)

A handful of PAs discussed retiring, and others were working towards this goal so had reduced the number of employers they worked with. Another PA was trying to retire but felt obligated to stay in the role until her employer’s family could find a replacement (which was proving difficult), having worked with their employer for a long time.

Several PAs described unmet demands for PAs in their localities:

*There is a huge demand for PAs*. *I scaled back my work during the last couple of months of last year*, *because I will be 66 this year and I do get really tired*. *So yes*, *they’re desperate for more PAs and the attrition rate is tremendous because it is very demanding*, *very badly paid*. (PA 93)

### PAs’ appetite for regulation ‐ ‘mixed feelings’

While there was agreement by PAs that they wanted to be more connected to and supported by LAs during the pandemic, there was reticence and little consensus about whether PAs wished to be professionally regulated as the following quote demonstrates:

*I’ve got mixed feelings about that because as soon as you have to register*, *you know*, *they start putting restrictions on you to an extent [] on the one hand*, *you know*, *things need regulating because they can go wrong*, *badly wrong*. *On the other hand*, *I love the freedom of this*. *You know*, *if you find somebody really nice that you can work with*, *there’s nobody breathing down your neck*. *Just have a trust relationship with the people*, *and it’s lovely*. *But also*, *there will be [] people who need protecting from carers*, *and carers who need protecting from employers who aren’t so good*. (PA 111)

Similar to the previous studies (88), PAs were in favour of DBS (Disclosure and Barring Service–checks of criminal records) checks being mandatory.

## Discussion

Findings have offered insights into the long-term effects of Covid-19 on the PA workforce in terms of: outsider status; support and training needs; job insecurity; and regulation debates.

A possible lasting impact of the pandemic is PAs’ strong identification as being an outsider workforce and of having been forgotten during the pandemic. A minority of PAs however felt that there had been some changes and they were now more visible to health and social care systems. Consideration should be given as to whether LAs should be required to have oversight of PAs employed via direct payments and whether there should be a LA point of contact for them, or whether brokerage services could independently deliver such support if comprehensively funded to do so.

A strong theme of the interviews was that PAs appreciated being connected to a supportive and pro-active broker service or LA organisation and having the opportunity to access information, peer knowledge-sharing/networking, training and potentially emergency support. This chimes with findings from Leverton et al., [26] who conducted interviews with disability support broker agency representatives outlining a range of services available during the pandemic *for PA employers*, such as information sharing, advocacy, befriending services or peer support hubs. These authors described how such services had been reduced or ceased due to austerity measures pre-Covid-19 and discussed how the pandemic had highlighted the need and potential for specific services to be commissioned to encourage joined up working between disability support organisations and local/central government.

The TLAP report [7] mentioned earlier, based on survey findings from 985 PA employers, also concluded: *“[LA] Commissioners need more quality assurance and better oversight of their local PA market*. *They should be also proactive in checking the quality*, *reliability and standard of support from those they commission to assist people supported by PAs*” (p7). PAs in our sample mentioned some tensions of working with broker organisations and lack of support, for example in disputes with employers. At the same time, this PA employers’ report found PAs were not able to afford to hire PAs via brokers or agencies due to the additional costs [7]. Highlighting the situation from an international perspective, a study from Australia [27], drawing on interviews with 28 organisational managers of disability support providers, noted difficulties reconciling competition, marketisation and multi-agency working in a direct payment marketplace.

The mental health implications of the pandemic were flagged up by PAs in our study as a support need for themselves, and also for PA employers. Policy makers have noted globally the need for mental health support for health care staff during and after the pandemic [28]. PA employers also raised this as an area where support was lacking [7]. An interview study of 40 private care home and home care workers in the UK highlighted triggers for mental health problems for staff, some of these resonate with narratives from PAs in our study, especially fear of infection and infecting others, lack of recognition compared to NHS staff, and lack of guidance [29].

Many PAs in our study were reportedly fairly satisfied with their access to training and new online opportunities available, which is a new development compared to our earlier study. Our previous study [20] found PAs saying they would appreciate specific condition-related training and they would be willing to take on more healthcare related tasks with appropriate training. Increased PA skills training for nursing tasks would therefore potentially enable employers to remain at home longer, reducing their risk of infection. We found a small amount of evidence that PAs had taken on more healthcare tasks during the pandemic. Further development of Personal Health Budgets (PHBs) (which are funded or partly funded by the NHS as well as social care) [30–32] could be considered as a way of assisting PAs to support employers to remain at home with more clinical oversight. Wilcock et al. [33] undertook interviews with 20 General Practitioners (GPs) and flagged up tensions between their appreciation of potentially valuable PA role and skills, while at the same time experiencing anxieties about establishing their identities and relationship to their client. GPs therefore called for increased skills training for PAs and the development of protocols for delegation of health tasks and safeguarding of vulnerable people. Wilcock et al. [34] also reported findings from interviews with 30 PAs about multi-disciplinary working and found taking on health liaison work, in place of family members, or care management or advocacy roles can be problematic unless PAs feel they are being respected by healthcare workers. Also striking a note of caution about encouraging PAs to undertake more healthcare tasks is Gousia et al.’s [35] study of PA recruitment and retention which re-analysed Skills for Care’s PA and PA employer survey data from 2017 and 2019 (n = 6,650); finding higher rates of PA vacancy/turnover when a PHB is the funding source for their employers. However, the complexity of this situation is underscored by Roland et al. [36] who examined the Skills for Care’s PA and Employers 2019 survey data (n = 2,428 PAs) and investigated the factors associated with PA absenteeism. They found an inverse relationship, that is, employers who had PHBs had PAs displaying lower rates of absenteeism. This situation was explained by the authors as potentially being due to employers in receipt of PHBs having higher healthcare needs and finding it difficult to find PAs that make a good match, leading to higher turnover rates. However, once a good match is found, the closer relationship between skillset and healthcare need results in lower rates of absenteeism. It was also suggested it could also indicate greater job satisfaction from greater training and skills development opportunities for a PA with an interest in health-related tasks.

An area of risk that has increased during the pandemic is staff shortages and consideration is needed to how more people can be encouraged to join the PA workforce. Similarly to our interviews, TLAP [7] highlighted the suffering of many PA employers during the pandemic and the safeguarding risks of their being left without support for long periods of time due to PA shortages. In the care sector overall Skills for Care [11] estimated an extra 480,000 people may be needed to work in social care in England by 2035 to keep pace with demand; if those aged 55 and over decide to retire, a further 430,000 people would be needed in the next 10 years. Most of our sample were aged in their 50s and 60s (mean 51 years) with 18% being over 65 and only 3% under 25 years. Compared to Skills for Care’s survey [10], where the mean age of participants was 42.3 years with 8% over 65 and 12% under 25 years, our sample was older (including 3 PAs who were 70 or over). Our sample highlighted PAs leaving the workforce due to retirement, health vulnerability (self or another family member) and seeking work where the risks of catching Covid-19 were less; in addition, most PAs mentioned poor pay and stress of the role. Care workers have one of the highest turnover rates in adult social care at 36.1% in 2021/22, up from 34.4% in 2020/21, however the turnover of PAs is reported to be lower according to a survey in which many were family members (18.3%) [11]. It may also be that PAs experience more emotionally rewarding relationships from their work. This may also explain the presence of difficulties experienced by PAs in maintaining boundaries [37], for example in our sample, some PAs wished to resign from their job, but felt obligated to remain in post as they did not want to leave employers in the lurch. These concerns are reflected internationally. For instance, the work of Sage et al. [38] in the United States on the impact of Covid-19 on PA employers and their unmet needs (although not looking directly at PAs) presents a parallel picture of dwindling recruitment within the PA workforce since Covid-19 and similarly notes low wages, high stress, and Covid-19-related fears as key factors. Similarly, in Ireland home care workers experienced similar problems to their English counterparts in terms of access problems to PPE and greater exposure to Covid-19 [39].

The importance of including PAs in infection control policies such as state funded schemes enabling PAs to isolate (or not lose pay due to restricting multi-site work), and have access to information, PPE and vaccines were raised in interviews. Skills for Care’s [40] survey noted care workers’ precarious working conditions (21% of PAs and 35% of care workers in the independent sector are on zero hours contracts) which potentially make decisions to take sick leave or self-isolate harder. TLAP [7] noted how PA employers felt their inability to pay PAs for sickness absence or isolation made recruiting a PA harder and PA employers wished to see the introduction of retention payments to allow them to keep paying PAs in these circumstances. From an international perspective, Reed et al. [16] analysed publicly available policy documents relating to financial support arrangements which included care workers across seven high-income international jurisdictions from March 2020 to March 2021. These authors compared access to financial support initiatives across countries demonstrating a range of options available to policy makers in planning for future emergencies.

Finally, a comparison of the professionalisation of the care workforce, including PAs, in the four UK nations [41] concluded that while increased regulation and mandatory training might be desirable and could be seen as professionalising, in the light of recruitment shortages in England, it could be unrealistic in the present circumstances. This chimes with our data which found many PAs to be reticent about more formal regulation, although interestingly keen for DBS checks to be mandatory which is a form of formal regulation.

### Strengths and limitations

Interviews in this study were carried out over the telephone or virtually rather than in person, with associated risks of missed interactional and contextual insights. As is common in such studies, PAs might be more likely to take part if they are legal residents, confident about their tax affairs, and, possibly, particularly committed to care work. There is a possibility that some did not want to recall their experiences during the pandemic. We were not able to interview all of the initial sample; some of the original participants were uncontactable, others declined the opportunity to participate for reasons of being too busy or for unstated reasons. This presents some risk of bias.

## Conclusions

Exploration of PA’s reflections over the pandemic period (collected from December 2021 to April 2022) has highlighted their perceptions of outsider status as directly-funded care workers, a reported lack of contact with LAs or wider health and care systems and a strong sense of a lack of support. The exceptions to this suggest that it is possible to reverse this position by funding intermediate organisations or for LAs to take on such responsibilities (as undertaken in one area by Hertfordshire Adult Care Services, 2021). Going forward, it is important to ensure PAs are considered in infection control and disaster/crisis planning nationally and locally, that notice is taken of their financial security, as well as considering ways they could be known to LAs, and their wider training needs and aspirations addressed.

## Supporting information

S1 Checklist COREQ (COnsolidated criteria for REporting Qualitative research) checklist. (PDF)
